# Preliminary human application of optical coherence tomography for quantification and localization of primordial follicles aimed at effective ovarian tissue transplantation

**DOI:** 10.1007/s10815-018-1166-9

**Published:** 2018-04-02

**Authors:** Seido Takae, Kosuke Tsukada, Ichiro Maeda, Naoki Okamoto, Yorino Sato, Haruhiro Kondo, Kiemi Shinya, Yuki Motani, Nao Suzuki

**Affiliations:** 1 Department of Obstetrics and Gynecology, St. Marianna University School of Medicine, 2-16-1 Sugao, Miyamae-ku, Kawasaki City, Kanagawa 216-8511 Japan; 2 Graduate School of Fundamental Science and Technology, Keio University, 3-14-1 Hiyoshi, Kouhoku-ku, Yokohama, Kanagawa 223-8522 Japan; 3 Department of Pathology, St. Marianna University School of Medicine, 2-16-1 Sugao, Miyamae-ku, Kawasaki City, Kanagawa 216-8511 Japan

**Keywords:** Optical coherence tomography, Ovarian tissue transplantation, Ovarian tissue cryopreservation, Ovarian reserve, Fertility preservation

## Abstract

**Purpose:**

The purpose of this study was to evaluate the possible clinical application of optical coherence tomography for assessing ovarian reserve in individual specimens of human ovarian tissue for fertility preservation.

**Methods:**

Ovarian tissue examination by optical coherence tomography was performed before ovarian tissue cryopreservation. Three of the four subjects had hematological disease or cancer, and they faced a threat to their fertility due to impending chemotherapy. One patient underwent ovarian tissue extraction for in vitro activation of dormant follicles as fertility treatment.

**Results:**

The current full-field optical coherence tomography technique can detect primordial follicles in non-fixed and non-embedded human ovarian tissue. These images are well correlated with histological evaluation and the ovarian reserve test, including follicle counts.

**Conclusion:**

It was demonstrated that optical coherence tomography could assess localization of primordial follicles and ovarian reserve in specimens of non-fixed human ovarian cortex, although optimization for examination of human ovarian tissue is needed for clinical application. Additionally, this technique holds the possibility of assessing the ovarian reserve of patients with unevaluable ovarian reserve.

**Trial registration number:**

UMIN000023141

## Introduction

Ovarian tissue cryopreservation has become the standard procedure for fertility preservation in young cancer patients who are facing loss of fertility due to impending chemotherapy and radiation therapy. According to the guidelines of the American Society of Clinical Oncology (ASCO) [1], although ovarian tissue cryopreservation is still an experimental and viable option for fertility preservation, more than 130 live births have already been reported [2–4]. Furthermore, in Germany, around 400 ovarian tissue cryopreservation procedures are carried out each year [5]. To date, more than 1500 ovarian tissue cryopreservation procedures have been performed in the Nordic countries [6]. In addition, two live births were reported applying the new clinical procedure of ovarian tissue transplantation (IVA: in vitro activation) as a fertility treatment for patients with primary ovarian insufficiency (POI) [7, 8]. Globally, the live birth rate from transplanted frozen-thawed ovarian tissue is approximately 30% [3, 5, 9, 10]. Although ovarian function is well recovered among most transplanted cases [3, 11], only 67–75% of patients had at least 1 year of ovarian tissue activity [10]. The duration of ovarian tissue activity normally depends on the number of remaining primordial follicles contained in the transplanted ovarian tissue. The main reasons for follicle loss are “burst” or “activation” of primordial follicle recruitment and ischemic apoptosis [3]. However, selection of ovarian tissue that includes a maximum number of primordial follicles is important for improving the outcome of ovarian tissue transplantation. Therefore, a procedure needs to be established for the assessment of ovarian reserve in ovarian tissue.

Optical coherence tomography (OCT) is a non-invasive imaging technique that provides high-resolution images for a range of clinical applications [12, 13]. By measuring back-scattered light from microstructural features in tissues, OCT images include micron-scale detail to a penetration depth of 1–3 mm [12, 14]. OCT has recently been used for the imaging of tissues for clinical examination of areas that can be accessed directly or by using an endoscope or catheter. OCT is used in such fields as ophthalmology [15, 16] and dentistry [17, 18], and to evaluate such areas of the body as the gastrointestinal tract [19, 20], coronary blood vessels [21, 22], colon [23], breast [24, 25], and more [12, 14]. Some researchers have been investigating the application of OCT in evaluating the anatomical features of normal ovarian tissue [13, 14, 26–29]. In particular, a study has been reported on the clinical application of OCT for the detection of malignant cells and to search for primordial follicles in formalin-fixed and embedded ovarian tissue for fertility preservation [30]. In addition, researchers have recently reported a study using mice ovaries to investigate the possibility of using OCT to assess ovarian reserve [31].

The present study investigated the possibility of using OCT to assess the localization of primordial follicles and ovarian reserve in ovarian tissue, using non-fixed human ovarian tissue that had been cryopreserved for fertility preservation due to POI and impending treatment of hematological disease and cancer.

## Material and methods

### Preparation of human ovarian tissue

Human ovaries were obtained from 4 patients who underwent ovariectomy for ovarian tissue cryopreservation between November 2013 and February 2017 for fertility preservation or fertility treatment at our university hospital. Ovarian tissue from Patients 1 and 2 was cryopreserved-thawed by vitrification methods using a commercial ovarian cryopreservation kit (Ova Cryo Kit Type M; KITAZATO Biopharma Co., Ltd., Shizuoka, Japan), as previously reported [7, 8]. The ovarian tissue from Patients 3 and 4 was fresh. A small (4–8 mm × 2 mm) 1-mm-thick section of ovarian cortex was cut out from part of each ovary. This process was performed in a petri dish (Bacteriological Petri Dish 60 × 15 mm, FALCON 351007; Becton, Dickinson and Company, Franklin Lakes, NJ, USA) containing modified HTF (mHTF) culture medium (with HEPES; KITAZATO Biopharma Co., Ltd.). For the present study, in accordance with the principles of the Declaration of Helsinki, all patients provided written, informed consent, and the study was approved by the institutional review board of our university (institutional approval No. 3311, UMIN000023141).

### Optical coherence tomography examination

The OCT system (Light-CT scanner; LL Tech, Paris, France) was used in the present study. The light source of this equipment is a halogen lamp. The mechanical characteristics of the Light-CT scanner included images with 1-μm resolution in all three dimensions that can be manipulated using a standard DICOM viewer. The wavelength of the OCT examination was 700–800 nm (near-infrared), the width of the wave was 125 nm, and the area of examination was 0.64 mm^2^. Images were obtained to a depth of 100 μm, which was the maximum depth at which high-resolution images could be taken. The mean exposure time during the OCT examination was approximately 2–3 min for each 0.64-mm^2^ section. The recorded images were modified using a Gaussian filter to reduce the speckle noise, and then contrast and brightness were adjusted to clarify the follicle boundaries. These modified images were reconstructed as representative images in accordance with histological images.

### Histological study of human ovarian tissue

The specimens of ovaries examined with OCT were fixed by 10% buffered formalin. The specimens were embedded in paraffin and sectioned into 4-μm-thick slices, placed on silane-coated glass slides, and stained with hematoxylin and eosin (H&E). OCT images were compared with H&E-stained histological images.

### Follicle counting

To verify the accuracy of OCT examination, the number of counted follicles was compared between OCT and histological imaging to a depth of 65 μm. In performing histological follicle counts on human ovaries, primordial follicles were defined as those containing flat follicular cells, and primary follicles were defined as those with one cuboidal follicular cell on histological study [32]. Follicles were counted only when the dark-staining nucleolus was seen within the nucleus of the oocytes to prevent recounting of the same follicle [33]. Meanwhile, when performing OCT follicle counts on human ovaries, a black dot of around 20–30 μm was regarded as a primordial and/or primary follicle. To avoid duplicate counting of follicles on OCT imaging, the black dots were counted carefully every 20 μm. With this OCT equipment, it is difficult to make an accurate distinction between primordial and primary follicles. Accordingly, the sum of primordial and primary follicles was compared between OCT and histological imaging. In this regard, it was difficult to count follicles in whole ovarian tissue due to the penetration of OCT (no more than 100 μm). Therefore, the follicles were counted on histological images after calculating the degree of shrinkage after fixation and paraffin embedding. Also, to reduce the bias of this process, follicle count was performed by three collaborators.

### Statistical analysis

JMP Pro version 12 (SAS Institute Inc., Cary, NC, USA) was used for statistical analysis. The numbers of follicles are expressed as means ± standard error (SE). The Wilcoxon signed-rank test was performed, and a *P* value < 0.05 was considered significant.

## Results

### Medical history and characteristics of the subjects

The characteristics of the patients are shown in Table 1. Patient 1 was 32 years of age with anal cancer. Her ovarian reserve was normal (a “normal ovarian reserve case”); her basal follicle-stimulating hormone (FSH) level was 4.34 mIU/ml, and her anti-Müllerian hormone (AMH) level was 5.01 ng/ml. Her ovarian tissue was cryopreserved for fertility preservation, but after her treatment for anal cancer, she became pregnant naturally.

**Table 1:** Characteristics of patients received ovarian tissue cryopreservation

Patient	1	2	3	4
Case	Normal OR	Menopausal OR	Unknown OR	Unknown OR
Disease/condition	Anal cancer	Severe DOR	Aplastic anemia	ALL
Age at OTC	32	45	15	11
Menstruation	Regular (30 days)	Primary amenorrhea	Before menarche	Before menarche
Basal FSH (mIU/ml)	4.34	20.1	6.0	3.0
AMH (ng/ml)	5.01	0.51 (before 1.5 year of ovariectomy)	1.99	0.07
Chemotherapy before OTC	―	―	―	CPA 3250 mg
Past history	Crohn disease	MRKH syndrome Unilateral ovarian cystectomy	―	―

*OTC* ovarian tissue cryopreservation, *FSH* follicle-stimulating hormone, *AMH* anti-Müllerian Hormone, *OR* ovarian reserve, *DOR* diminished ovarian reserve, *ALL* acute lymphocytic leukemia, *CPA* cyclophosphamide, *MRKH syndrome* Mayer-Rokitansky-Kuster-Hauser syndrome

Patient 2 (a “menopausal case”) was 45 years of age and had severely diminished ovarian reserve (DOR). She underwent ovariectomy for IVA. Her basal FSH level was 20.1 mIU/ml, and her AMH level was 0.51 ng/ml at 1.5 years prior to ovariectomy. This patient had primary amenorrhea and was diagnosed with Mayer-Rokitansky-Küster-Hauser syndrome (MRKH syndrome) because she had a hypoplastic uterus. Although she received ovarian tissue transplantation for IVA, she could not become pregnant. The cryopreserved-thawed ovarian tissues of patients 1 and 2 were investigated by OCT.

Patients 3 and 4 were investigated as “unknown ovarian reserve cases.” These patients had not yet experienced menarche. Patient 3 was 15 years of age with aplastic anemia, and she underwent ovarian tissue cryopreservation before bone marrow transplantation. Her basal FSH level was 6.0 mIU/ml, and her AMH level was 1.99 ng/ml. Patient 4 was 11 years of age with acute lymphatic leukemia; she underwent ovarian tissue cryopreservation before bone marrow transplantation. In addition, she received chemotherapy (3250 mg of cyclophosphamide) before consultation for fertility preservation. Her basal FSH level was 3.0 mIU/ml, and her AMH level was 0.07 ng/ml. In these teenaged patients, basal FSH and AMH levels are not reliable markers for assessing real ovarian reserve.

### Comparison between OCT images and histological images of human ovarian tissue

#### Patient 1 (normal ovarian reserve case)

**Fig. 1:**
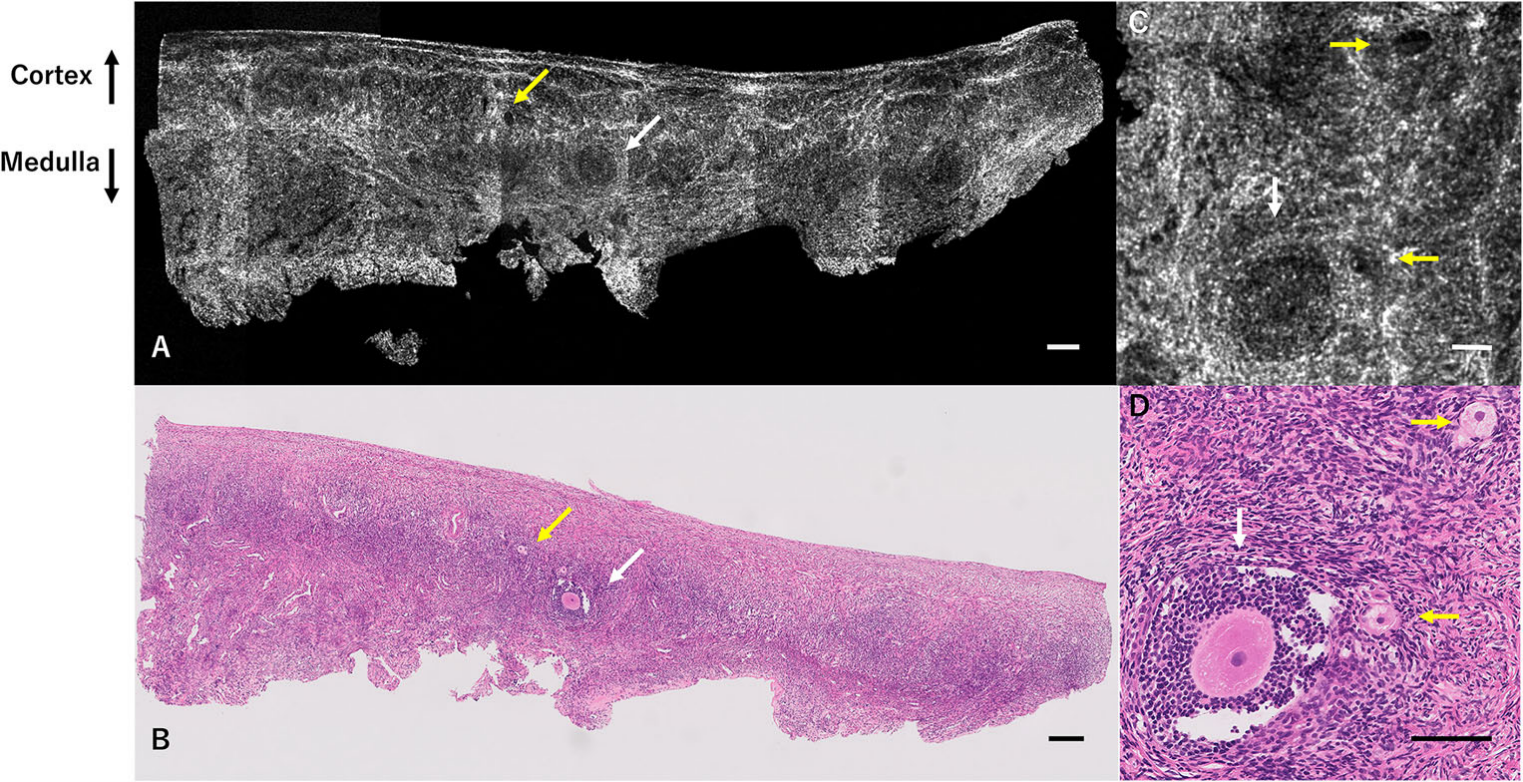
OCT images and H&E-stained histological images of ovarian tissue from patient 1 (normal ovarian reserve case). Results of OCT examination of ovarian tissue from patient 1, who was 32 years of age with anal cancer. Several developing follicles (primordial-primary: yellow arrows, and antral follicles: white arrows) are detected by OCT (**a**), in accordance with histological examination (**b**). This OCT image reflects the normal result of the ovarian reserve test (FSH 4.34 mIU/ml, AMH 5.01 ng/ml). Magnified OCT image (**c**) demonstrates the details of developing follicles (primordial-primary: yellow arrows, and antral follicles: white arrows) in accordance with magnified histological examination (**d**). Scale bar = 200 μm (**a**, **b**), 100 μm (**c**, **d**). *OCT* optical coherence tomography, *H&E* hematoxylin and eosin, *FSH* follicle-stimulating hormone, *AMH* anti-Müllerian hormone

Figure 1a–d shows a set of OCT images and H&E-stained histological images from patient 1. The OCT images show several follicles including primary-primordial and antral follicles, in accordance with the H&E-stained histological images.

#### Patient 2 (menopausal case)

**Fig. 2:**
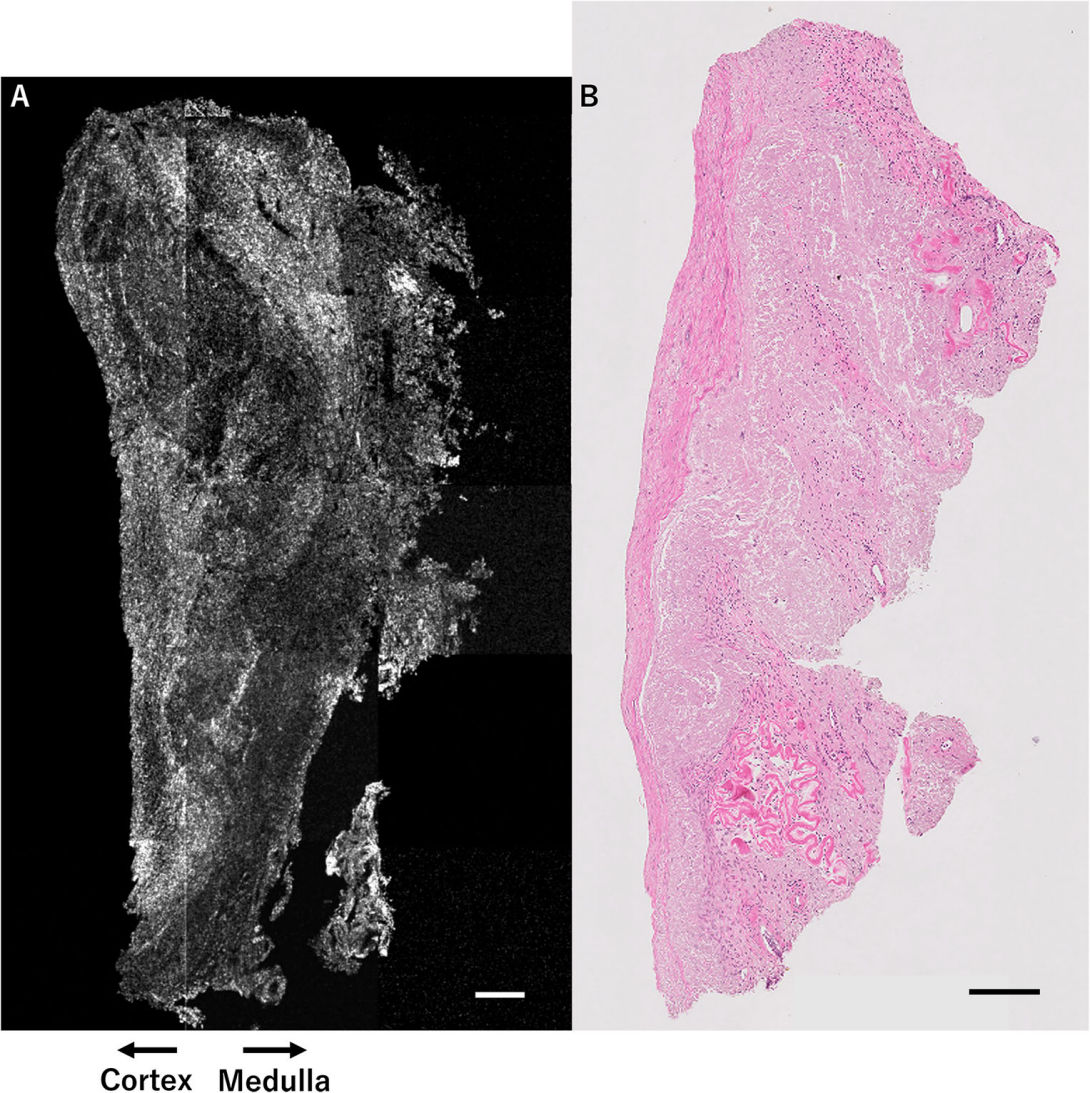
OCT image and H&E-stained histological image of ovarian tissue from patient 2 (menopausal case). Results of OCT examination of ovarian tissue from patient 2, who was 45 years of age with severely diminished ovarian reserve. No follicles are detected by OCT (**a**), in accordance with histological examination (**b**). This OCT image reflects the poor result of the ovarian reserve test (FSH 20.1 mIU/ml, AMH 0.51 ng/ml at 1.5 years prior to ovariectomy). Scale bar = 200 μm (**a**, **b**). *OCT* optical coherence tomography, *H&E* hematoxylin and eosin, *FSH* follicle-stimulating hormone, *AMH* anti-Müllerian hormone

Figure 2a, b shows a set of OCT images and H&E-stained histological images from patient 2. No follicles were detected by OCT, in accordance with the H&E-stained histological images. These images represent very low ovarian reserve, corresponding with basal FSH and AMH levels.

#### Patients 3 and 4 (unknown ovarian reserve cases)

**Fig. 3:**
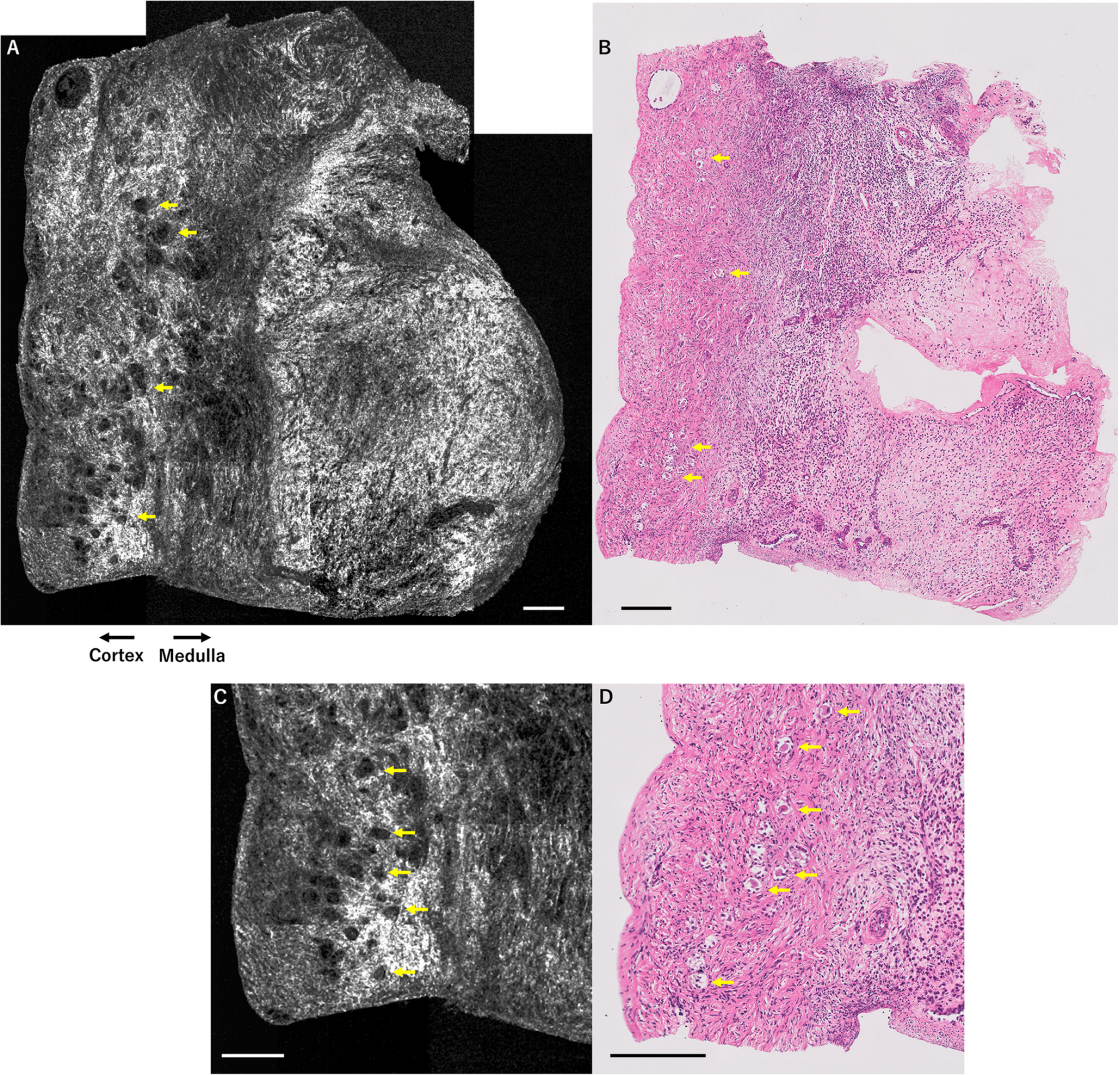
OCT images and H&E-stained histological images of ovarian tissue from patient 3 (unknown ovarian reserve case). Results of OCT examination of ovarian tissue from patient 3, who was 15 years of age with aplastic anemia. Her AMH and basal FSH levels were unevaluable for ovarian reserve assessment because she was premenarcheal. Several primordial-primary follicles (yellow arrows) are detected by OCT (**a**), in accordance with histological examination (**b**). Magnified OCT images (**c**) demonstrate the details of primordial-primary follicles (yellow arrows) in accordance with magnified histological examination (**d**). Scale bar = 200 μm (**a**–**d**). *OCT* optical coherence tomography, *H&E* hematoxylin and eosin, *FSH* follicle-stimulating hormone, *AMH* anti-Müllerian hormone

**Fig. 4:**
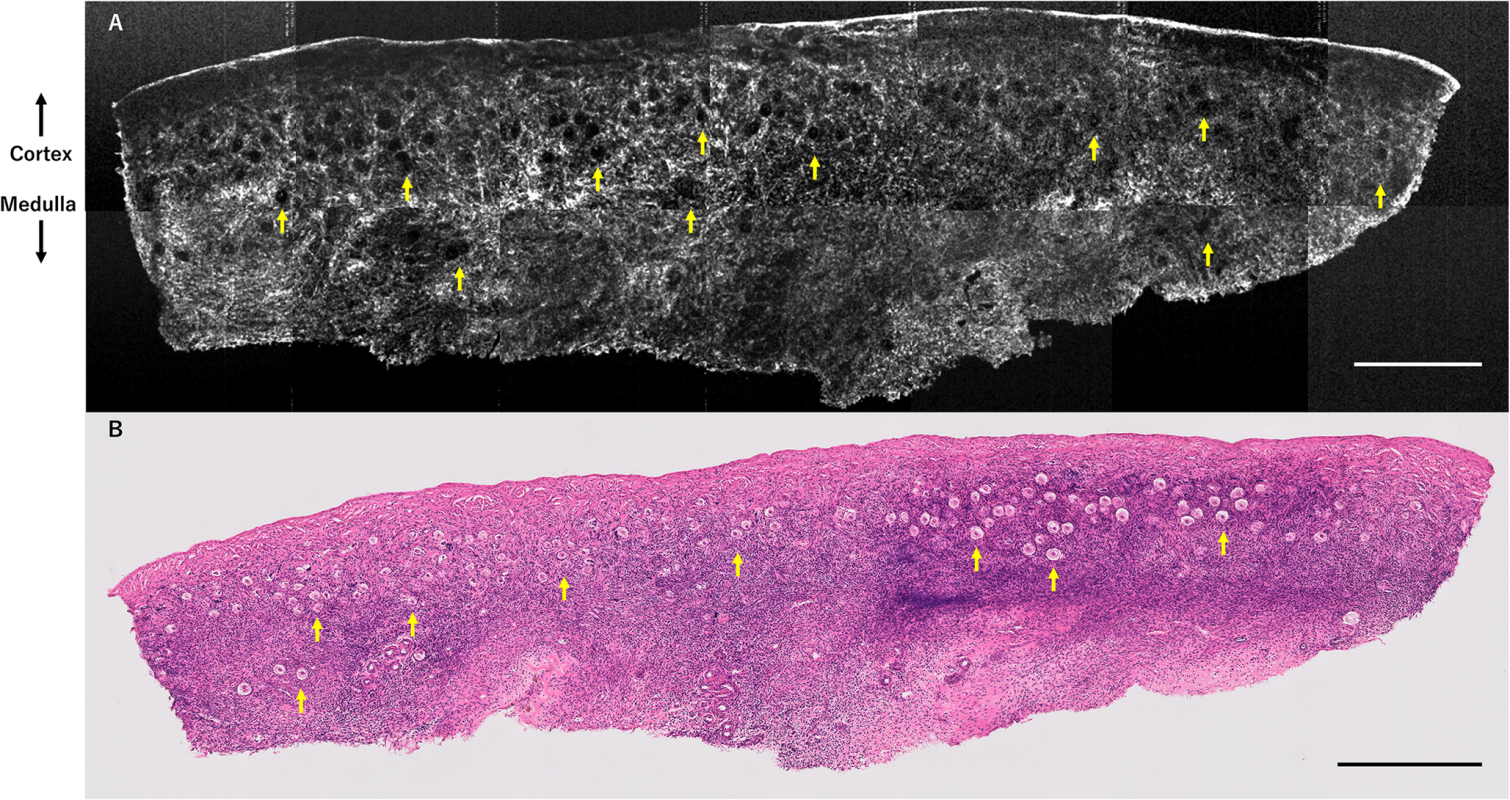
OCT image and H&E-stained histological image of ovarian tissue from patient 4 (unknown ovarian reserve case). Results of OCT examination of ovarian tissue from patient 4, who was 11 years of age with acute lymphatic leukemia. Although her AMH and basal FSH levels were unevaluable for ovarian reserve assessment because she was premenarcheal and in the midst of a chemotherapy treatment cycle, several primordial-primary follicles (yellow arrows) are detected by OCT (**a**), in accordance with histological examination (**b**). Scale bar = 500 μm (**a**, **b**). *OCT* optical coherence tomography, *H&E* hematoxylin and eosin, *FSH* follicle-stimulating hormone, *AMH* anti-Müllerian hormone

Figures 3a–d) and 4a, b show a set of OCT images and H&E-stained histological images from patients 3 and 4. Although their AMH levels were low (patient 3, 1.99 ng/ml; patient 4, 0.07 ng/ml), many primordial follicles were detected in the ovarian cortex of both patients.

On pathological examination, malignant cells were not detected in the ovarian tissues of patients 1 through 4.

Meanwhile, there were no differences among fresh (patients 1, 2) and vitrified-thawed (patients 3, 4) ovarian tissue in terms of image quality and appearance of primordial-primary follicles.

### Accuracy of follicle counts using OCT

**Fig. 5:**
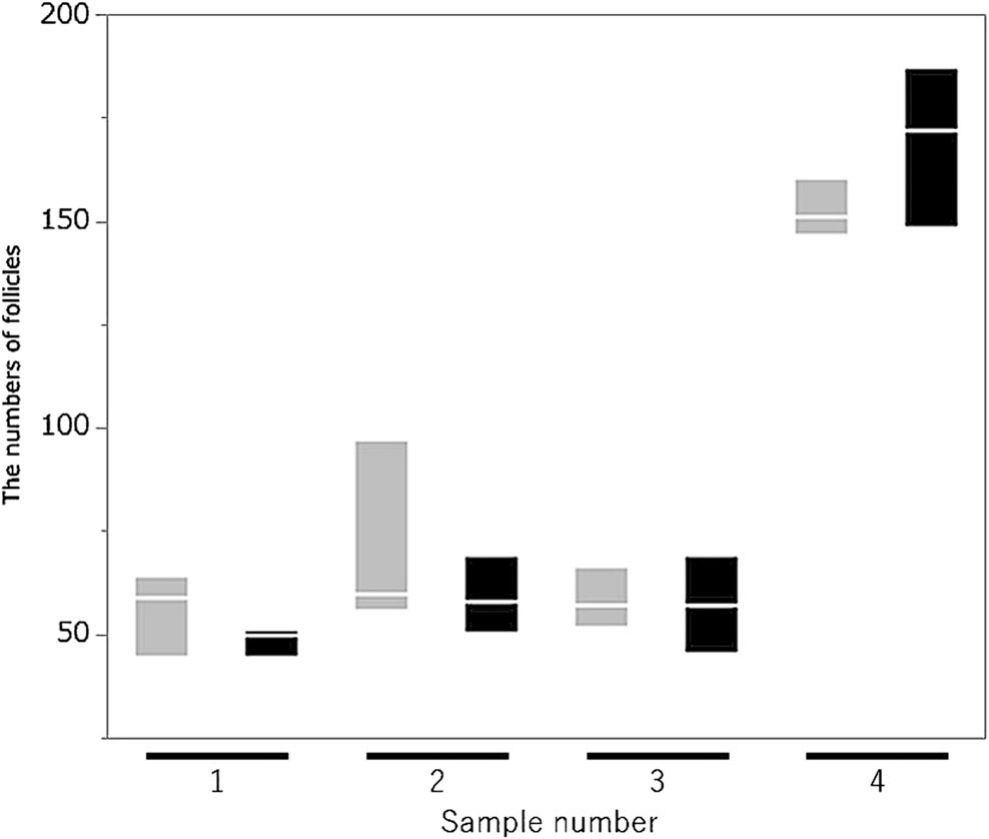
A comparison of follicle counts between OCT and histological examination. There is no significant difference in follicle counts between OCT and histological examination (*P* = 0.78, Wilcoxon signed-rank test). Sample number 1–3: patient 3, 4: patient 4. Gray boxplot: OCT examination, Black boxplot: histological study

On the follicle counting of four serial ovarian sections obtained from girls with hematological disease and before menarche, the means of the sum of primordial and primary follicles of ovarian tissue were 84.5 ± 43.0 (OCT) and 83.7 ± 52.9 (histology); there was no significant difference between OCT and histological evaluation (*P* = 0.78). Also, Fig. 5 shows the follicle counts for OCT and histological imaging (sample number 1–3: patient 3, sample number 4: patient 4).

## Discussion

### Advantage of OCT for assessment of the numbers of primordial follicle intend for effective ovarian tissue transplantation

Comparison of OCT imaging and H&E-stained histological images demonstrated that OCT could detect primordial follicles clearly. Detection of primordial follicles on formalin-fixed and embedded human ovarian tissue was reported in 2016 [30]; this was the first report of detection of primordial follicles in fresh human ovarian tissue with clear OCT images. In 2017, researchers reported the detection of primordial follicles in fresh murine ovaries [31]. Through non-invasive detection of primordial follicles in fresh human ovarian tissue, localization of primordial follicles can be assessed. Primordial follicles have been shown to be very unevenly distributed in the cortex of ovaries [34]; these findings indicate that OCT can assist in selecting the most productive ovarian tissue for ovarian tissue transplantation. The present study is the first case series of OCT examination using clinical samples intended for assessment of the quantity and localization of primordial follicles as the first step for clinical application.

OCT images were compared between patient 1 (normal ovarian reserve case) and patient 2 (menopausal case), and the outcomes were correlated with H&E-stained histological images and the ovarian reserve test (basal FSH and AMH levels). Although present study is the first report that showed certain accuracy of OCT, and indicated correlation with basal FSH and AMH levels, at present, the accuracy of OCT compared with histological examination remains debatable [31]. Although OCT examination does not have any advantage in terms of image quality compared with histological studies, the biggest advantage of OCT examination is that there is no need for any fixation and paraffin embedding, unlike conventional histological studies. This means we can transplant ovarian tissue whose follicle count has already been evaluated. We believe that the ovarian tissue selection may improve the efficacy of ovarian tissue transplantation, because primordial follicles were very unevenly distributed throughout the ovarian cortex [34]. As a matter of course, more investigation is needed to confirm the correlation between the numbers of counted follicles using OCT and ovarian function after ovarian auto-transplantation, although such confirmation is very difficult with human fresh ovaries because the testable depth of high-resolution OCT still extends only to 100 μm [31]. Nevertheless, to apply OCT in clinical medicine, establishment of a follicle count system in conjunction with OCT, such as an automatic cell counter and a statistical computing method, is needed. A rapid screening and rapid counting system would be vital for the effective practice of clinical medicine.

### Development and modification of OCT for primordial follicle detection

Exciting developments have occurred in OCT. The first generation of OCT was time-domain OCT (TD-OCT), which can be used to compose an image that is acquired by point-by-point scanning of both the interferometer reference arm length and the laser beam. Subsequently, frequency-domain (Fourier-domain) OCT was developed, which replaced the reference arm length scan with parallel spectroscopic measurements (spectral-domain OCT) or sequential measurements made by scanning the wavelength of a tunable laser (swept-source OCT). Full-field OCT differs from TD-OCT and frequency-domain OCT in that it does not use a scanning light beam to produce tomographic images in the *en face* orientation (orthogonal to the optical axis). Unlike with conventional OCT, low spatial coherence light illuminates the entire field of the image [35]. The present study used a full-field OCT system to pursue rapid image creation and high-resolution imaging. Some improvements of the OCT technique (e.g., a wavelength or scanning system) are needed to investigate human ovarian tissue for assessment of actual follicle localization and ovarian reserve. Indeed, OCT technology has evolved rapidly, and attempts have been made to achieve ultra-high-speed imaging with a modified luminous source and a high scanning rate system [36, 37]. Methods of speckle noise reduction (e.g., Gaussian filtering) for improvement of image quality have been investigated [38, 39]. These investigations may allow OCT to move closer to practical use. Although the present study was preliminary, it indicated the possibility of clinical application. Certainly, histological study is superior in terms of accuracy for ovarian reserve assessment, though OCT has advantages in terms of swiftness and non-invasiveness, although it is still under development. Further, OCT is a unique method for assessing follicle localization and ovarian reserve without fixation, dyeing, or contact.

### Utility of OCT to assess the ovarian reserve for patients with unevaluable ovarian reserve

In the present study, OCT was applied effectively for patients who had unknown ovarian reserve (patients 3 and 4). These patients were prepubertal and premenarcheal, so the serum FSH level was not reliable for assessing the ovarian reserve of these patients. Additionally, AMH levels are low during prepubertal development, rise during early puberty, and reach a plateau at 20–25 years of age [40, 41]. Serum AMH levels decline immediately after initiation of chemotherapy and increase after several months of chemotherapy, but no markers attain the pretreatment values [42]. In particular, patient 4 received chemotherapy (3250 mg of cyclophosphamide) before consultation for fertility preservation; therefore, the AMH level was not reliable for assessing her ovarian reserve. Although supplementary data of the ASCO guidelines has set a high-risk POI dosage for cyclophosphamide treatment [1], the innate amount of ovarian reserve varies between individuals. Consequently, the total dosage of cyclophosphamide is not a conclusive factor in determining the ovarian reserve of a patient. However, assessment of the ovarian reserve of such patients is greatly needed to evaluate the efficacy of ovarian tissue cryopreservation and transplantation. Unfortunately, at present, there is no procedure to accurately assess ovarian reserve and distribution, as with histological examination. With optimization of inspection of the ovary, OCT examination could assess the ovarian reserve of child patients and chemotherapy patients who have low AMH levels, which indicate a seemingly low ovarian reserve.

### Further investigation of OCT for fertility preservation

To accurately assess fresh ovarian reserve by OCT, several issues need to be resolved regarding OCT equipment. One issue is testable depth; as mentioned above, the current testable depth of high-resolution OCT reaches only 100 μm. The existing model of OCT equipment does not readily support testable depth and resolution simultaneously. Second, modification of the method for examining human ovarian tissue is needed. In the present study, ex vivo OCT examination was performed on dissected ovarian cortex. To assess ovarian reserve before ovarian tissue cryopreservation, in vivo OCT examination is needed, for example, integrated with endoscopy or laparoscopy or a catheter [21, 22, 26]. Implementation of in vivo laparoscopic or catheter examination for ovarian reserve assessment and a faster acquisition rate and downsizing are also needed while maintaining high resolution [26]. Third, the scanning area is too shallow to obtain OCT imaging of the ovary. The scanning area of the OCT equipment used in the present study was 0.64 mm^2^. Expansion of the scanning area would be important for clinical application. In the area of ophthalmological research, megahertz OCT for ultrawide-field retinal imaging with a 1050-nm Fourier-domain mode-locked laser has been reported [43], having seven times the width of the current scanning area and 200 times the scanning speed of the conventional system.

Ideally, we hope to investigate the use of OCT for assessing ovarian reserve from the surface of the body as with ultrasonography by high-speed, real-time image processing. This would be a truly non-invasive and effective method for assessment of ovarian reserve and primordial follicle localization. Additionally, further safety assessments are needed for clinical application of OCT; only one paper mentioned the reproductive safety of OCT technique using mice, and there are no reports to date that mentioned human reproductive safety [30]. In addition, more investigation is needed to confirm the “non-invasiveness for reproductive cells” of OCT examination through evaluation of follicle viability after NIR irradiation, although a report indicated its non-invasiveness using a glucose uptake assay and neutral red staining with bovine ovarian tissue [30]. Further verification of the non-invasiveness for reproductive cells under various study conditions (wavelength, illuminance, irradiation time, and energy) will bring OCT closer to clinical application in reproductive medicine.

## Conclusion

In the present study, the possibility of the clinical application of OCT for assessment of the localization of primordial follicles and ovarian reserve was demonstrated. OCT could also assess ovarian reserve in patients with unevaluable ovarian reserve. Although clinical applications of OCT are not feasible with the current technology, the optimization and improvement of OCT equipment will make it possible to select the most suitable ovarian tissue containing abundant follicles for the purpose of successful ovarian tissue transplantation. As a next step, it will be necessary to compare the effectiveness of OCT imaging and conventional methods for ovarian tissue selection.
